# Quantitative Foveal Structural Metrics as Predictors of Visual Acuity in Human Albinism

**DOI:** 10.1167/iovs.65.3.3

**Published:** 2024-03-05

**Authors:** Erica N. Woertz, Gelique D. Ayala, Niamh Wynne, Sergey Tarima, Serena Zacharias, Murray H. Brilliant, Taylor M. Dunn, Deborah Costakos, C. Gail Summers, Sasha Strul, Arlene V. Drack, Joseph Carroll

**Affiliations:** 1 Department of Cell Biology, Neurobiology and Anatomy, Medical College of Wisconsin, Milwaukee, Wisconsin, United States; 2 School of Medicine, Medical College of Wisconsin, Milwaukee, Wisconsin, United States; 3 Department of Ophthalmology and Visual Sciences, Medical College of Wisconsin, Milwaukee, Wisconsin, United States; 4 Division of Biostatistics, Institute for Health and Equity, Medical College of Wisconsin, Milwaukee, WI, United States; 5 Center for Precision Medicine Research, Marshfield Clinic, Marshfield, Wisconsin, United States; 6 Department of Genetics, University of Alabama at Birmingham, Birmingham, Alabama, United States; 7 Department of Ophthalmology and Visual Sciences, University of Iowa Hospitals and Clinics, Iowa City, Iowa, United States; 8 Department of Ophthalmology and Visual Neurosciences, University of Minnesota, Minneapolis, Minnesota, United States

**Keywords:** albinism, foveal hypoplasia, OCT, visual acuity

## Abstract

**Purpose:**

To assess the degree to which quantitative foveal structural measurements account for variation in best-corrected visual acuity (BCVA) in human albinism.

**Methods:**

BCVA was measured and spectral domain optical coherence tomography (SD-OCT) images were acquired for 74 individuals with albinism. Categorical foveal hypoplasia grades were assessed using the Leicester Grading System for Foveal Hypoplasia. Foveal anatomical specialization (foveal versus parafoveal value) was quantified for inner retinal layer (IRL) thickness, outer segment (OS) length, and outer nuclear layer (ONL) thickness. These metrics, participant sex, and age were used to build a multiple linear regression of BCVA. This combined linear model's predictive properties were compared to those of categorical foveal hypoplasia grading.

**Results:**

The cohort included three participants with type 1a foveal hypoplasia, 23 participants with type 1b, 33 with type 2, ten with type 3, and five with type 4. BCVA ranged from 0.08 to 1.00 logMAR (mean ± SD: 0.53 ± 0.21). IRL ratio, OS ratio, and ONL ratio were measured in all participants and decreased with increasing severity of foveal hypoplasia. The best-fit combined linear model included all three quantitative metrics and participant age expressed as a binary variable (divided into 0–18 years and 19 years or older; adjusted *R*^2^ = 0.500). This model predicted BCVA more accurately than a categorical foveal hypoplasia model (adjusted *R*^2^ = 0.352).

**Conclusions:**

A quantitative model of foveal specialization accounts for more variance in BCVA in albinism than categorical foveal hypoplasia grading. Other factors, such as optical aberrations and eye movements, may account for the remaining unexplained variance.

Human albinism is characterized by foveal hypoplasia,^1^^,^^2^ decreased foveal cone density,^1^^,^^3^ abnormal decussation of optic nerve fibers at the optic chiasm,^4^^,^^5^ and aberrant organization of visual space in visual cortical areas.^6^^,^^7^ Affected individuals commonly experience nystagmus, decreased or absent depth perception, and decreased best-corrected visual acuity (BCVA).^8^ These anatomical and clinical features vary widely in severity between affected individuals, so there is interest in clarifying the relationship between visual system structure and function in albinism. Reduced BCVA is the visual feature that has the greatest impact on affected individuals’ lifestyles and will be an important outcome measure for novel therapeutics.^9^^,^^10^ Additionally, accurate estimates of future BCVA at the time of diagnosis (which often occurs when children are too young to provide subjective acuity measurements) may assist patients and their families to manage expectations for life-long visual function.

Foveal hypoplasia has proven to be a strong structural predictor of BCVA in albinism.^11^^,^^12^ The normal fovea is characterized by several key anatomical specializations, including lateral displacement of inner retinal layers (IRL, which forms the characteristic pit), elongation of cone outer segments (OS), and thickening of the outer nuclear layer (ONL) relative to the parafovea. In foveal hypoplasia, inner retinal layers persist over the incipient pit, and OS elongation and ONL thickening may also be decreased or absent.^2^^,^^13^ The severity of foveal hypoplasia is commonly evaluated subjectively using the Leicester System for Grading Foveal Hypoplasia (hereafter referred to as the “Leicester System”), which assigns a categorical grade based on the presence or absence of each anatomical specialization.^12^ These grades correlate well with BCVA in albinism,^2^^,^^11^^,^^12^ supporting foveal hypoplasia severity as a predictor for functional outcomes.^12^ However, there is considerable overlap in the range of visual acuities observed within each foveal hypoplasia grade.^2^^,^^12^ This suggests that additional structural features not captured in a categorical grading system may also contribute to BCVA. One possibility is that quantitative structural metrics might better describe the wide spectrum of foveal specialization in albinism^14^^–^^16^ and allow more precise functional predictions. A recent study found that quantitative foveal measurements did not predict BCVA as well as categorical grade,^12^ but the quantitative metrics in that study were limited to the fovea itself rather than measuring foveal structure *relative* to the parafovea. Additionally, retinal thickness varies widely within the normal population,^17^ so this normal variation may have confounded accurate staging of foveal maturity.

Here, we examine a quantitative approach to assess retinal structure in human albinism that normalizes the foveal thickness of the IRL, OS, and ONL relative to the parafoveal thickness. We find that there is greater variability in foveal structure among individuals with albinism than is indicated by categorical grades. We show that quantitative metrics, when combined with participant demographics, explain more of the variance in BCVA in albinism than categorical foveal hypoplasia grading. Although categorical grading remains an important and clinically-accessible tool for patient care, our findings suggest that quantitative metrics may also be valuable as prognostic indicators of visual function.

## Methods

### Participants

All experiments adhere to the tenets of the Declaration of Helsinki and were approved by the Medical College of Wisconsin Institutional Review Board (PRO 23898). All participants, or participants’ legal guardians if under 18 years old, provided informed consent before collection of research data. The initial cohort included 90 participants who were confirmed, likely, or suspected to have albinism (see *Albinism Classification* below) and excluded any participants with retinal pathology other than albinism. All participants completed an Ocular Health Questionnaire and axial length was measured using an IOL Master (Carl Zeiss Meditec, Dublin, CA, USA). Monocular BCVA or visual acuity with correction was measured during a clinic visit or with Early Treatment Diabetic Retinopathy Study charts.^18^

### Albinism Classification

Genetic testing was performed using a blood or saliva sample as previously described.^3^^,^^15^ Test results were reviewed for variants in the *TYR*, *OCA2*, *TYRP1*, *SLC45A2*, *SLC24A5*, and *GPR143* genes, which correspond to oculocutaneous albinism type 1 (OCA1), OCA2, OCA3, OCA4, and OCA6 and ocular albinism type 1 (OA1), respectively. Results were also reviewed for variants in Hermansky-Pudlak syndrome genes. See Supplementary Appendix 1 for criteria used to determine variant pathogenicity.

Participants were classified as having confirmed albinism if they had at least two out of three clinical criteria (nystagmus, ocular hypopigmentation [as evidenced by iris transillumination or macular hypopigmentation], and foveal hypoplasia.)^8^^,^^19^^–^^25^ and a confirmed genetic diagnosis. Participants with likely albinism were those with all three clinical criteria but only one albinism-causing variant in an OCA or Hermansky-Pudlak syndrome gene, and participants with suspected albinism were those with all three clinical criteria but no albinism-causing variants in any of the examined albinism genes.

### Bioptigen OCT Image Acquisition and Processing

Adult participants’ pupils were dilated with phenylephrine hydrochloride (2.5%) and tropicamide (1%). Children were dilated with Cyclomydril (1% phenylephrine hydrochloride and 0.2% cyclopentolate). OCT images were acquired at the incipient fovea using a Bioptigen spectral domain OCT (Leica Microsystems, Wetzlar, Germany). Both volume scans and horizontal line scans were acquired; line scans contained 80 to 120 frames and were nominally 5 to 8 mm in length.

OCT scans for each subject were processed by a single observer (E.N.W.) using ImageJ.^26^ First, the volume scan was reviewed to determine which frame(s) corresponded to the center of the incipient fovea. This determination was based on features such as minimum inner retinal layer thickness, maximum outer segment (OS) elongation, or maximum outer nuclear layer (ONL) thickness, as previously described.^2^^,^^12^^,^^13^ Next, the line scan was reviewed to confirm that it captured the incipient fovea; if not, then a single foveal frame from the volume scan was used for further analysis. If the line scan did capture the incipient fovea, then one frame was chosen as a reference to register all remaining frames using the TurboReg plugin.^27^ The registered frames were reviewed and manually deleted if they did not accurately register to the reference frame. The registered frames (no more than 19) were averaged to generate the final processed image. If none of the other frames in the line scan accurately registered to the reference, then the single reference frame was used for further analysis.

The axial scale of each image (µm/pixel) was determined using the manufacturer specifications of the original OCT scan. To determine the lateral scale of each image, the length of each scan was calculated by multiplying the nominal scan length by the ratio of the participant's axial length to the assumed axial length of the OCT device (24 mm). This scan length was then divided by the number of A-scans per B-scan used for acquisition (1000).^3^ All processed line scans were then resampled using the bicubic automatic algorithm in Photoshop CS6 (Adobe, San Jose, CA, USA) to a final axial scale of 2.27 µm/pixel and a lateral scale of 5.26 µm/pixel.

### Foveal Hypoplasia Grading

Processed Bioptigen OCT scans were used to subjectively assess each participant's categorical foveal hypoplasia grade according to the Leicester System.^12^ All scans were graded independently by two observers (E.N.W. and N.W.). For scans where the two observers disagreed, they discussed the foveal features and arrived at a consensus grade, which was used as the final foveal hypoplasia grade.

### Quantitative Metrics of Foveal Structure

We used three metrics—OS ratio, IRL ratio, and ONL ratio—to quantify the amount of OS elongation, IRL lateral displacement, and ONL thickening (respectively) present at the incipient fovea. Thus these metrics correspond to the same foveal specializations that determine categorical foveal hypoplasia grading in the Leicester System. Each metric was designed to measure the thickness of the specified retinal layer *relative to the parafovea* and is expressed as a ratio, such that a higher number represents a greater degree of structural specialization. The methods used to calculate these ratios are described in detail below.

### Outer Segment Measurements

The length of the photoreceptor OS was defined as the distance between the ellipsoid zone (EZ, also referred to as the inner segment/OS junction) and the interdigitating zone (IZ, also referred to as the cone OS tips) and was measured using longitudinal reflectivity profiles (LRPs). Processed Bioptigen line scans were loaded into OCT Reflectivity Analytics (ORA) software^14^ and converted to a linear format for display purposes.

Maximum outer segment length was calculated by a single observer (E.N.W.) as previously described.^14^ The location of the apparent maximum OS length was first subjectively identified by the observer, then LRPs (five pixels wide) were calculated every 25 µm within 250 µm of the apparent maximum, generating a total of 21 LRPs. The peaks corresponding to the EZ and IZ in each LRP were labeled by the user and exported from ORA. Using Matlab (Mathworks, Natick, MA, USA), a single-term Gaussian function was fit to the peak-to-peak distances between the EZ and IZ as a function of retinal location. The maximum of the fitted function was used to calculate both the maximum OS length and the location of the incipient fovea in each scan.

After determining the location of the maximum OS length, the OS elongation ratio (hereafter referred to as the “OS ratio”) was calculated. First, the average parafoveal OS length was calculated in ORA by placing a single LRP (five pixels wide) 1.75 mm from the fovea both nasally and temporally, then averaging the peak-to-peak distances between the EZ and IZ from the temporal and nasal sides (with two exceptions: for KS_10314 and JC_12537, the OS length at 1.75 mm was only measurable on one side). This location (1.75 mm) was chosen based on the method used by McAllister et al.,^1^ because OS length is relatively stable beyond this eccentricity. The OS ratio was calculated as the maximum OS length divided by the average parafoveal OS length.

### Inner Retinal Measurements

Processed Bioptigen OCT scans were loaded into the Duke OCT Retinal Analysis Program (DOCTRAP)^28^ for automatic segmentation. The “Normal” algorithm segments nine retinal layers: the inner limiting membrane (ILM), retinal nerve fiber layer (RNFL), combined ganglion cell layer (GCL) and inner plexiform layer (IPL), inner nuclear layer (INL), outer plexiform layer (OPL), external limiting membrane (ELM), EZ, IZ, and retinal pigment epithelium (RPE). Automatic segmentation was manually corrected for errors. The segmentation coordinates, in which each layer includes a *y*-coordinate at every pixel along the *x*-axis of the image, were then exported from DOCTRAP for further calculations.

The inner retinal layer (IRL) thickness was defined as the distance between the ILM and the INL/OPL boundary (which includes the RNFL, GCL, IPL, and INL) and was calculated by multiplying the distance between the ILM and INL/OPL boundary (in pixels) by the axial scale of the image (2.27 µm/pixel). The location of the maximum OS length (as calculated above) was used as the foveal center. The IRL thickness was smoothed using a 30-pixel moving average then linearly interpolated at 100-µm increments. The IRL ratio was calculated as the average IRL thickness at 1 mm from the fovea (average of nasal and temporal thickness) divided by the foveal IRL thickness. This eccentricity (1 mm) was chosen because this is where the GCL and IPL reach their maximum thickness (on average) among individuals with albinism.^16^

### Directional OCT Processing & Outer Nuclear Layer Measurements

For a subset of participants (n = 55) directional OCT (D-OCT) images were also acquired using a Cirrus high-definition OCT (Carl Zeiss Meditec) as previously described.^15^^,^^29^^,^^30^ This method was chosen because it enables ONL measurements that are independent of the Henle fiber layer (HFL). D-OCT was not performed for all participants because successful D-OCT requires high image quality and consistency between successive scans; thus it was attempted only in participants who had less severe nystagmus and demonstrated good image quality on other OCT scans. Cirrus D-OCT images for each participant were aligned as previously described,^15^^,^^29^^,^^30^ and a maximum-intensity image was created in ImageJ.^26^ D-OCT scans from 24 participants included in final analyses.

The merged maximum-intensity D-OCT images were segmented by a single observer (E.N.W.) as previously described.^15^ Briefly, the merged image and the two individual directional scans were loaded into a stack in ImageJ,^26^ and the multi-point tool was used to segment the ILM, OPL, HFL, ELM, EZ, and IZ with 30 points per layer. Although the merged image was the one ultimately segmented, the user referred to the variable reflectivity of the HFL in the directional images to assist with point placement for the OPL and HFL contours. For the 50 participants who did not have acceptable D-OCT images, a single Cirrus or Bioptigen line scan was used to segment the HFL so that ONL measurements were completed for all participants. The coordinates of all points were exported and custom Matlab software was used to calculate ONL thickness as previously described,^30^ then all thickness measurements were linearly interpolated to a common set of eccentricity coordinates (every 50 µm within 2 mm of the fovea). The location of the fovea was manually selected by a single observer (E.N.W.) by choosing the location of maximum distance between the ONL and IZ. The ONL ratio for each participant was then calculated as the ONL thickness at the fovea divided by the average ONL thickness at 1.75 mm eccentricity (average of nasal and temporal thickness).

### Statistical Analysis

To assess interobserver agreement, we calculated a weighted Cohen's κ using Cicchetti-Allison weights.^31^ To test whether data were normally distributed, we used the Shapiro-Wilk test; data were classified as normal if *P* > 0.05. Normal data are summarized in the text using mean and standard deviation (SD), and non-normal data are summarized using median and interquartile range (IQR). To test whether quantitative metrics varied with subjective foveal hypoplasia grade, we performed either a one-way ANOVA or the Kruskal-Wallis test and then used either the Holm-Sidak or Dunn's multiple comparisons test to assess pairwise comparisons between groups post-hoc (with tests chosen based on normality). To test whether quantitative metrics of foveal structure can objectively differentiate between categorical foveal hypoplasia grades, we performed receiver operating characteristic (ROC) analyses in which the subjective grade was ground truth. We assessed the sensitivity and specificity of IRL ratio to differentiate between grades 1a–1b and 2–4 (i.e., those with and without a foveal depression), that of OS ratio to differentiate between grades 1a–2 and 3–4 (i.e., those with and without OS elongation), and that of ONL ratio to differentiate between grades 1a-3 and 4 (i.e. those with and without ONL thickening). The optimum cutoff was selected as the IRL ratio, OS ratio, or ONL ratio with the highest sum of sensitivity and specificity. The Shapiro-Wilk test, ANOVA, Kruskal-Wallis test, Tukey multiple comparisons test, Dunn's multiple comparisons test, and ROC analyses were performed using Prism 9 software (GraphPad Software, Inc., La Jolla, CA, USA).

To test whether quantitative metrics of foveal structure can predict BCVA more accurately than a categorical foveal hypoplasia grade, we used linear regression modeling. We used a single predictor regression to evaluate categorical foveal hypoplasia grade as a predictor of BCVA. Next, we used a multiple regression analysis to build a predictive model that considered IRL ratio, OS ratio, ONL ratio, and participant age and sex (as well as their interactions) as predictors of BCVA. We used an iterative stepwise variable selection process to identify a linear model that provided the best predictive accuracy, which was not required to include all parameters. Goodness of fit of each multiple linear regression was evaluated using adjusted *R*^2^ values, which were adjusted based on the number of predictors included in each model. Linear models were generated using R (The R Foundation, Vienna) and validated using a “leave one out” cross-validation approach. The difference between predicted and observed BCVA for all participants was analyzed using the method described by Bland and Altman.^32^^,^^33^

## Results

### Participant Characteristics and Genotypes

All participant characteristics are summarized in Supplementary Table S1. Ninety participants with albinism completed OCT imaging and visual acuity testing, including 45 (50.0%) with confirmed albinism, 32 (35.6%) with likely albinism, and 13 (14.4%) with suspected albinism. There were 44 males (48.9%) and 46 females (51.1%). Participant ages ranged from five to 75 years with a median (IQR) of 18 (12–29) years.

Genetic analysis revealed seven coding variants in the cohort that were presumed to be albinism-causing and, to the authors’ knowledge, have not previously been reported (Supplementary Table S2): three in *TYR*, one in *OCA2*, one in *TYRP1*, one in *SLC45A2*, and one in *GPR143*. Additionally, one novel upstream variant was identified (AD_11940, *TYR* c.-4586_del4461bp_insTT) that was presumed to be albinism-causing because of likely disruption of the *TYR* gene promoter. Each of these novel variants was counted as albinism-causing when determining participants’ genetic diagnoses.

Sixteen participants were excluded from further analysis of retinal images because of poor OCT image quality. Final analyses included 74 eyes (44 right, 30 left) from 74 participants, including 36 (48.6%) with confirmed albinism, 28 (37.8%) with likely albinism, and 10 (13.5%) with suspected albinism. Of these, 38 participants (51.4%) were between five to 18 years old, and 36 participants (48.6%) were 19 years old or older. Among the included eyes, axial length (mean ± SD) was 22.82 ± 1.55 mm.

### Categorical Foveal Hypoplasia Grade and BCVA

When two observers (E.N.W. and N.W.) used the Leicester scale to assign categorical grades for foveal hypoplasia, they agreed on 52 images (70.3%) and disagreed on 22 images (29.7%). This corresponds to substantial interobserver agreement (weighted Cohen's *κ* = 0.695). Most cases of disagreement were between grades 2 and 3 (n = 15), in which observers disagreed about whether OS elongation was present. Other cases of disagreement were between 1b and 2 (n = 3, presence/absence of a foveal depression), between 3 and 4 (n = 2 presence/absence of ONL thickening), or between 2 and 4 (n = 2). Observers reached agreement in all cases of initial disagreement after reviewing foveal specializations together. The cohort included 74 participants with final grades of 1a (n = 3), 1b (n = 23), 2 (n = 33), 3 (n = 10), and 4 (n = 5).

**Figure 1.**
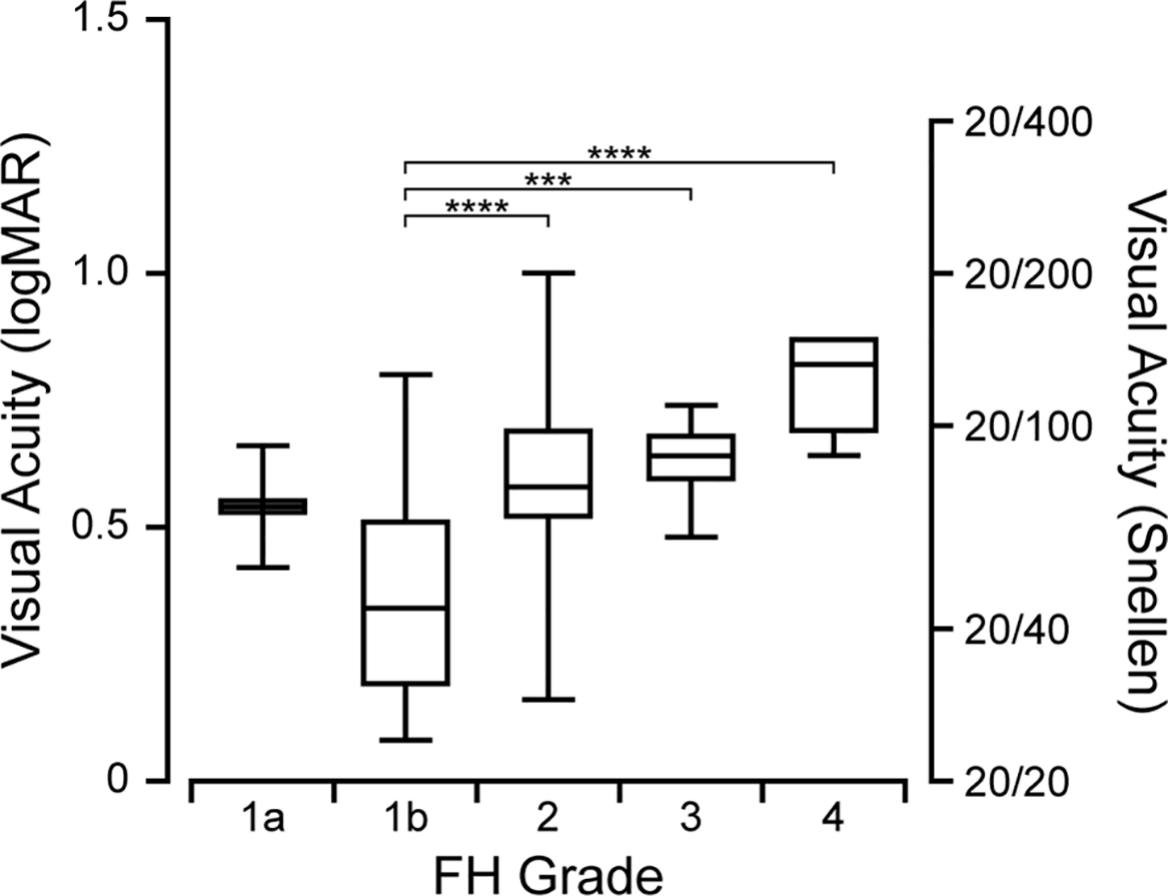
BCVA is generally worse with increasing subjective severity of foveal hypoplasia (FH), but there is considerable overlap between FH grades. *Brackets* indicate pairs of groups for which the Holm-Sidak multiple comparisons test showed a difference with *P* < 0.001 (***) or *P* < 0.0001 (****).

The BCVA (expressed as logMAR, or logarithm of the minimum angle of resolution) among all participants ranged from 0.08-1.00, with a mean (± SD) of 0.53 ± 0.21. Mean (± SD) BCVA was 0.54 ± 0.12 for participants with grade 1a, 0.36 ± 0.19 for grade 1b, 0.58 ± 0.18 for grade 2, 0.63 ± 0.08 for grade 3, and 0.79 ± 0.11 for grade 4. As shown in Figure 1, participants with more severe foveal hypoplasia had worse BCVA (one-way ANOVA test, *P* < 0.0001).

### Quantifying Foveal Specialization

**Figure 2.**
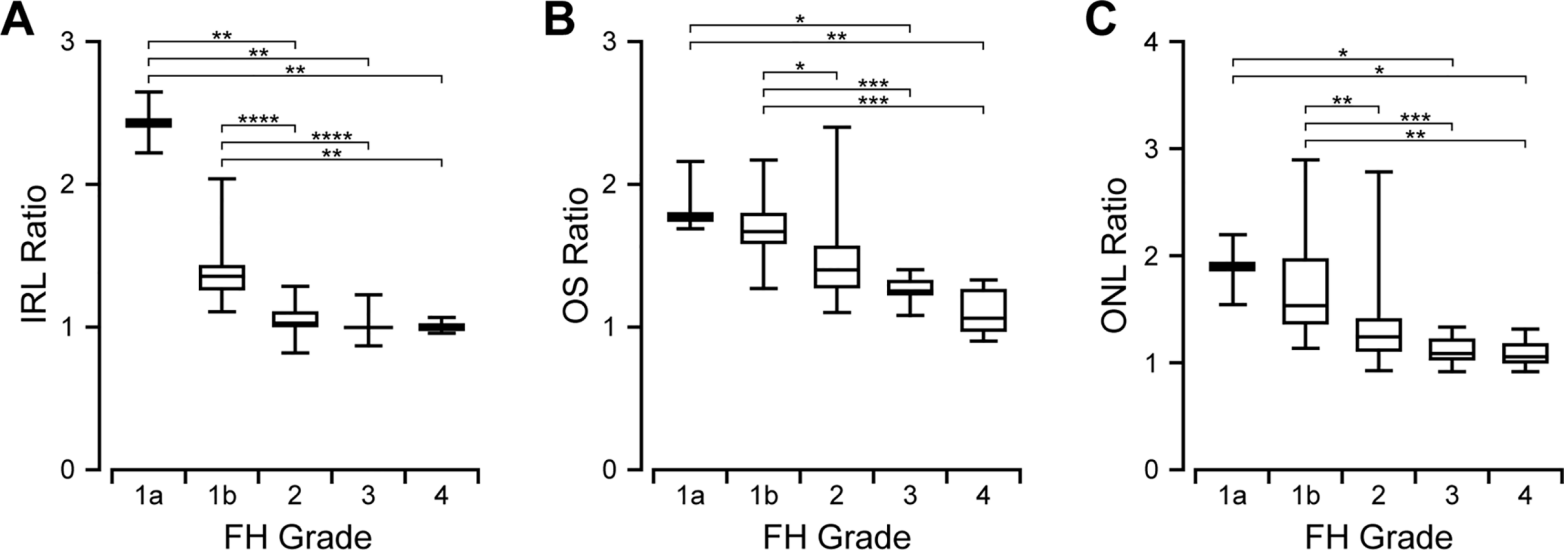
IRL ratio (**A**), OS ratio (**B**), and ONL ratio (**C**) all decrease with increasing severity of categorical foveal hypoplasia (FH) grade. However, each quantitative metric overlaps considerably between FH grades that were assigned according to the Leicester system. Box limits show interquartile range; *horizontal bars* within boxes show median; *whiskers* show minimum and maximum. *Brackets* indicate pairwise comparisons for which Dunn's multiple comparisons test shows a difference with *P* < 0.05 (*), *P* < 0.01 (**), *P* < 0.001 (***), or *P* < 0.0001 (****).

**Figure 3.**
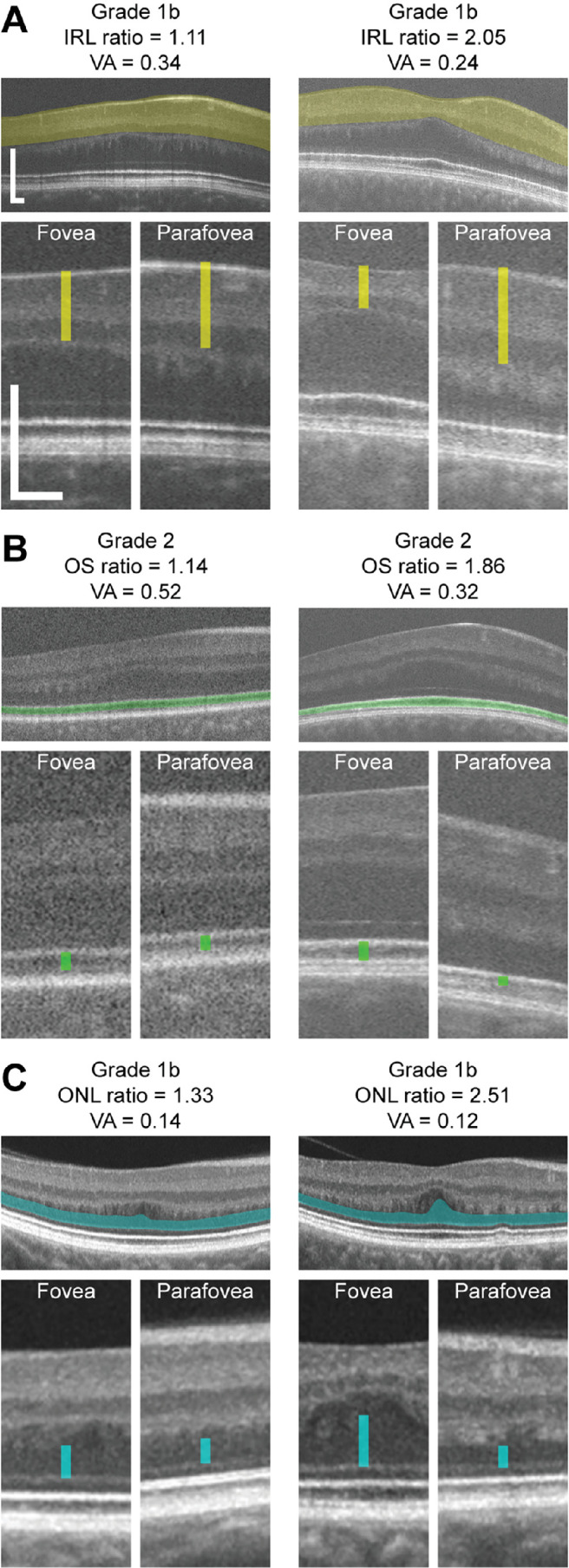
Foveal specialization and visual acuity (VA) varies between individuals with the same categorical grade of foveal hypoplasia. Shown are OCT scans with callouts of the foveal and parafoveal regions from pairs of participants who had the same foveal hypoplasia grade (as assigned according to the Leicester system) but highly disparate IRL ratio (**A**), OS ratio (**B**), or ONL ratio (**C**). Colored bars indicate the foveal and parafoveal IRL thickness (*yellow*), OS length (*green*), and ONL thickness (*cyan*). Parafoveal regions are from the nasal retina at the eccentricity (relative to the foveal center) where each measurement was taken (**A**, 1 mm; **B** and **C**, 1.75 mm). *Scale bars*: 200 µm.

The IRL ratio, OS ratio, and ONL ratio values for participants with each subjective foveal hypoplasia grade are summarized in Figure 2. These metrics were designed to quantify the amount of IRL lateral displacement (IRL ratio), OS elongation (OS ratio), and ONL thickening (ONL ratio) that is present at the fovea relative to the parafovea, such that a higher number represents greater structural specialization. Overall, the IRL ratio ranged from 0.82–2.66 (median = 1.10), OS ratio ranged from 0.90–2.40 (median = 1.46), and ONL ratio ranged from 0.90–2.89 (median = 1.27). IRL ratio, OS ratio, and ONL ratio all decreased with increasing foveal hypoplasia grade (Kruskal-Wallis test; IRL ratio: *P* < 0.0001; OS ratio: *P* < 0.0001; ONL ratio: *P* < 0.0001). All three metrics overlapped considerably between categorical grades. This was especially true for OS ratio: most notably, the range of values observed in grades 3 and 4 (images in which OS elongation was subjectively “absent”) significantly overlapped with those observed in grade 2 (images in which OS elongation was subjectively “present”). Similarly, for ONL ratio the range of values observed in grade 4 (in which ONL thickening was subjectively “absent”) was nearly identical to those observed in grade 3 (in which ONL thickening was subjectively “present”), and both grades 3 and 4 overlapped with grade 2. In contrast, the ranges of IRL ratios observed for grades 1a and 1b (images in which a foveal depression was subjectively “present”) showed better separation from those observed in grades 2–4 (images in which a foveal depression was subjectively “absent”). There was a wide range of specialization present within categorical grades, especially grade 1b (Fig. 3).

### Comparing Predictive Models of BCVA

**Figure 4.**
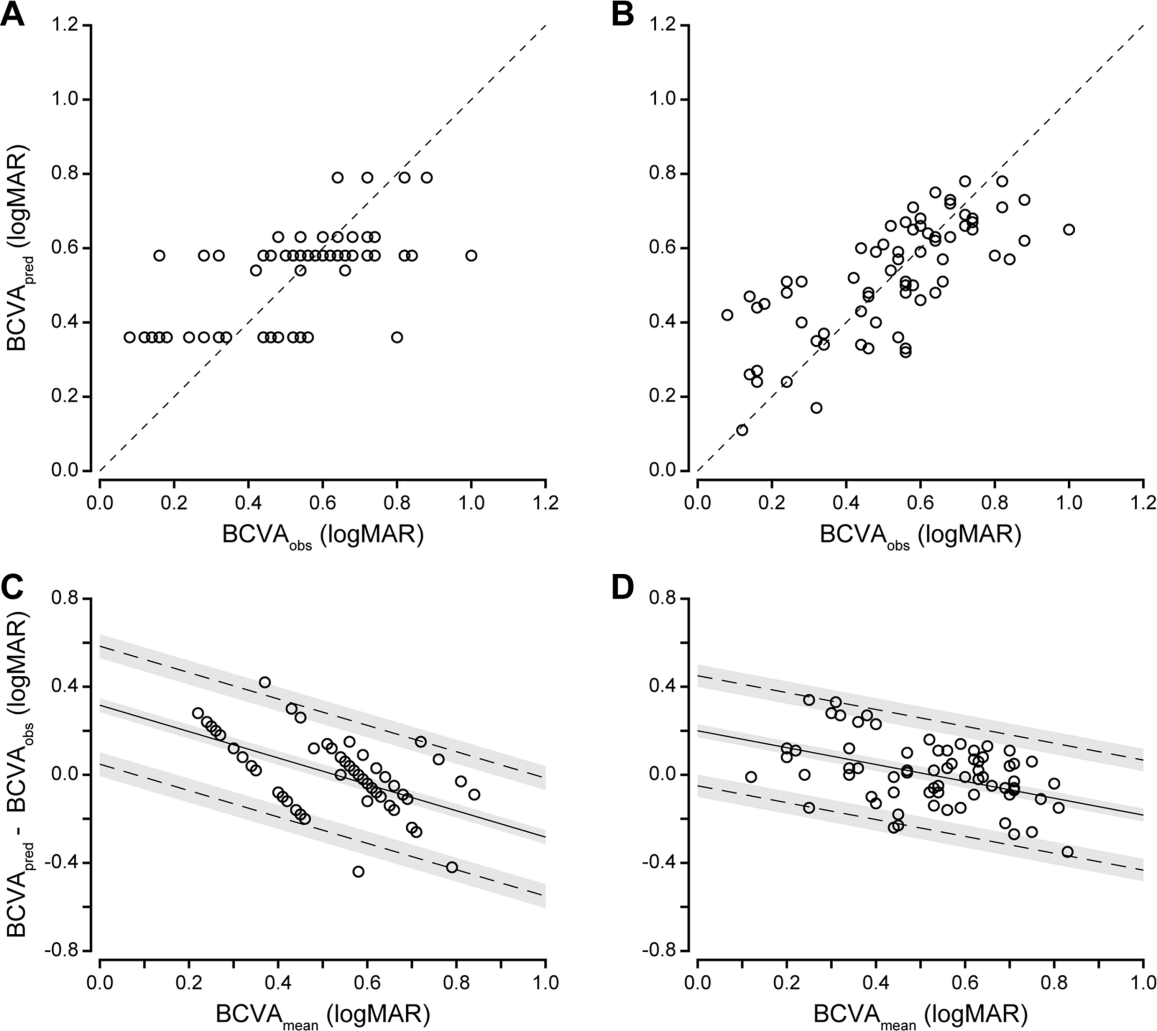
A combined linear model based on quantitative metrics of foveal structure predicts BCVA in albinism more accurately than categorical foveal hypoplasia grade. The correlation between predicted BCVA (BCVA_pred_) and observed BCVA (BCVA_obs_) was moderate for the predictive model based on categorical foveal hypoplasia grade (**A**, *R* = 0.622), but was stronger for the combined linear model based on quantitative structural metrics (**B**, *R* = 0.736). In panels **A** and **B**, *dashed lines* show where BCVA_pred_ = BCVA_obs_ (i.e., perfect agreement between predicted and observed BCVA). When we plotted the difference between predicted and observed BCVA (BCVA_pred_ – BCVA_obs_) against the average of predicted and observed BCVA (BCVA_mean_), there was a negative trend in the bias for both the categorical predictive model (**C**) and the combined linear model (**D**); however, this trend was reduced in the combined linear model. In panels **C** and **D**, *solid lines* show bias, *dashed lines* show limits of agreement, and *shaded gray areas* show the 95% confidence intervals of the bias and limits of agreement.

We examined two predictive statistical models of BCVA as a function of foveal structure: a single predictor regression based on categorical foveal hypoplasia grade, and a multiple regression based on quantitative metrics of foveal anatomical specialization. The goodness of fit for each model is shown in the Table. When using categorical foveal hypoplasia grades to predict BCVA, the model fit was moderate (*R*^2^ = 0.352). This model accurately predicted BCVA within 0.3 logMAR in 93.2% of participants and within 0.2 logMAR in 78.4% of participants. The correlation between predicted and observed BCVA was moderate (*R* = 0.622, Fig. 4A).

**Table.** Cross-Validated Goodness of Fit for Each Predictive Model

Predictive Model	Adjusted *R*^2^	RMSE	Correlation (*R*) Between Predicted and Observed BCVA
Categorical grade	0.352	0.170	0.622
Combined linear model	0.500	0.158	0.736

RMSE, root mean squared error.

When IRL ratio, OS ratio, ONL ratio, and participant sex and age were combined into a linear regression model, the model with the highest adjusted *R*^2^ and best predictive accuracy included all three quantitative metrics (IRL ratio, OS ratio, and ONL ratio). It also included participant age expressed as a binary variable, where age 18 is equal to 0 for participants 18 years old or younger, or equal to 1 for participants 19 years or older. This binary age variable was determined post-hoc to account for differences between children and adults. The model is expressed in the following equation:

$$\begin{array}{ccc} & & \;logMAR_{pred}=1.128-0.076*\left(ONL\;ratio\right)\\ & & \;+\;0.396*\left(Age\;18\right)+0.131*\left(IRL\;ratio\right)\\ & & \;-\;0.449*\left(OS\;ratio\right)-0.579*\left(Age\;18\right)*\left(IRL\;ratio\right)\\ & & \;+\;0.206*\left(Age\;18\right)*\left(OS\;ratio\right) \end{array}$$

This combined linear model fit the data (adjusted *R*^2^ = 0.500) better than the predictive model based on foveal hypoplasia grade. It accurately predicted BCVA within 0.3 logMAR in 94.6% of participants and within 0.2 logMAR in 79.7% of participants. The correlation between predicted and observed BCVA for this model (*R* = 0.736, Fig. 4B) was superior to that of categorical foveal hypoplasia grade.

When we used a Bland-Altman analysis^32^^,^^33^ to examine the discrepancy between predicted and observed BCVA for the predictive model based on categorical grade (Fig. 4C), there was a significant trend in the bias (linear regression: *y* = −0.574*x* + 0.305, *F* = 29.65, *DFn* = 1, *DFd* = 72, *P* < 0.0001). This indicates that categorical grade underestimates logMAR for participants with poorer BCVA and overestimates logMAR for participants with better BCVA. This was also true for the combined linear model (Fig. 4D; linear regression: *y* = −0.352*x* + 0.188, *F* = 15.51, *DFn* = 1, *DFd* = 72, *P* = 0.0002). The slope of the trend was closer to zero and the limits of agreement of the bias were slightly narrower for the combined linear model (±0.250 logMAR) than for the predictive model based on categorical grade (± 0.268 logMAR). However, these differences were not statistically significant.

## Discussion

### Predicting Visual Acuity From Foveal Structure

In this study we present quantitative metrics to objectively assess IRL lateral displacement, OS elongation, and ONL thickening in foveal hypoplasia. We find that IRL ratio, OS ratio, and ONL ratio can effectively identify these foveal structural specializations with high sensitivity and specificity (Supplementary Appendix 2). We also show that a quantitative statistical model of foveal structure can predict BCVA in albinism more accurately than categorical foveal hypoplasia grade. This quantitative model accounts for 50% of the variation in BCVA in our cohort.

This finding differs from previous studies for two reasons. First, we found foveal hypoplasia grade was less predictive of BCVA than previously reported.^12^ One reason is that prior observations were in a mixed group including albinism, idiopathic infantile nystagmus, and achromatopsia. Our study is more homogenous, including only individuals with albinism. Another reason may be differences in subjective grading criteria.^11^^,^^12^ Our data suggest that, consistent with previous findings,^14^ OS elongation and ONL thickening have many gradations between “present” and “absent.” These features were more ambiguous for observers to assess subjectively than presence of the foveal pit. OS ratio and ONL ratio also showed more quantitative overlap between categorical foveal hypoplasia grades than IRL ratio (Figs. 2B, 2C, 3B, 3C). Additionally, ROC analyses showed worse agreement between subjective and objective evaluation of OS elongation, or ONL thickening than that of foveal pit formation (Supplementary Appendix 2). The wide variation in OS elongation could also explain why we report more participants with grade 2 foveal hypoplasia than grade 3, whereas other studies with similar cohort sizes and study designs report greater incidence of grade 3 than grade 2.^2^^,^^12^ This may reflect a difference not in cohort composition but rather in the subjective threshold that different observers use to discriminate between the presence and absence of OS elongation. Such differences likely contributed to the imperfect agreement between observers when assigning foveal hypoplasia grades in our study. This is important for future studies because one of the goals for novel therapeutics for albinism is to improve foveal development,^10^ but this outcome cannot be accurately assessed unless there is consistency in grading conventions between observers and study sites.

The second reason our results differed was improved predictive power of our quantitative model, likely because of novel metrics. Many previously-proposed metrics—including foveal developmental index (the photoreceptor length divided by the total retinal thickness at the fovea),^12^^,^^34^ OS length,^12^^,^^14^ and photoreceptor length^12^—measure the thickness of specific layers only at the fovea, which fails to take into account their thinning or thickening *relative* to the periphery. This is important not only in the context of foveal hypoplasia^2^ but also when measuring foveal structure in individuals with normal retinae, because normal variation in retinal thickness because of genetic background could confound direct comparisons in absolute thickness measurements between individuals.^17^ When we examined these other metrics in our own population, we found a similar result as prior studies: foveal developmental index and photoreceptor length did not predict BCVA as accurately as categorical grade (Supplementary Appendix 3). Interestingly, OS length alone was a slightly better predictor than categorical grade, consistent with prior observations that OS length correlates well with BCVA in both albinism and aniridia.^13^^,^^35^ By expressing foveal structural specialization as a ratio of parafoveal structure, the metrics presented in this study both capture *relative* foveal specialization and normalize all thickness measurements for expected variation in retinal thickness between participants.

### Building a Quantitative Model

When building the linear model, we considered including IRL ratio, OS ratio, ONL ratio, and participant age and sex, but ultimately we selected parameters based entirely on their ability to improve the linear model's predictive power; the approach was agnostic to any theoretical assumptions about each parameter. The final model included all three quantitative metrics and participant age. OS ratio as a significant contributor to visual acuity predictions agrees with prior studies^13^^,^^35^ (as noted above), although the inclusion of IRL ratio and ONL ratio was more surprising. Prior observations suggest that the foveal pit itself has little influence on visual acuity.^3^^,^^11^^,^^13^^,^^36^^–^^39^ Similarly, ONL thickening is often assumed to develop as a result of foveal cone packing, which has previously been shown to correlate poorly with visual acuity in albinism.^3^ However, in the context of foveal hypoplasia, it is possible that IRL lateral displacement and ONL thickening serve as indirect markers of other developmental processes pertinent to visual acuity, as discussed below. The inclusion of all quantitative metrics also supports a hypothesis proposed by Rufai et al.^12^: when considering that singular quantitative metrics predict visual acuity less accurately than categorical grade, the authors suggested “the [categorical] grading system incorporates all key elements of foveal development, whereas [each quantitative metric] represents only individual developmental landmarks.” Thus incorporating IRL ratio, OS ratio, and ONL ratio into a single model provides a better prediction of visual acuity than any single metric alone.

Participant age was also included in the linear model, and it improved the model more when it was expressed as a binary variable (0–18 vs. 19 and older). This may reflect how visual acuity varies more during childhood than adulthood, both in albinism and in normal visual development.^38^ Participant sex was not included, suggesting that the known variability in retinal thickness caused by biological sex^17^ makes little contribution to visual function in this population.

### Other Factors Contributing to Visual Acuity in Albinism

Our combined linear model based on quantitative metrics accounts for more of the variance in BCVA in albinism than categorical foveal hypoplasia grade, but its predictive power remains imperfect. The *R*^2^ for this model was 0.500, accounting for only half of the variance in the data. It is possible that foveal morphology is not itself the main driver of BCVA, but rather that it indicates the presence of other cellular changes that play a more direct role.^40^ For example, some studies suggest that private line circuitry between foveal cones and their ganglion cell synaptic partners may be disrupted in albinism,^16^ which would be consistent with both the observed reduction in BCVA^41^ and the presence of foveal hypoplasia.^37^ Additionally, a recent study by Malechka et al.^42^ indicates that nystagmus and refractive error may also contribute to visual acuity predictions. Ultimately, a statistical model that accounts for these additional features of albinism would likely perform better than both categorical grade and the combined linear model we present here. Deep learning techniques such as those employed by Malechka et al.^42^ may be helpful to incorporate all these features into a unified model.

### Limitations

This study included measurements only from horizontal line scans, not vertical scans. Recent evidence indicates that retinal layer topography can differ between the horizontal and vertical axes.^16^ The high frequency of severe nystagmus and high refractive error also negatively impacted image quality, leading to the exclusion of many participants from the final analyses. In addition, the metrics we present here are not as clinically accessible as categorical grading. We designed our image acquisition and processing protocols to obtain the best-quality images possible in the study population, but these techniques are labor-intensive and generally impractical for clinical use. However, future development of novel software and hardware tools may enable automation of some of these steps in order to mitigate the notable difference in time and effort required to obtain quantitative versus categorical measurements.^12^^,^^43^^,^^44^

The participant cohort included only those with confirmed, likely, or suspected albinism, but there are numerous retinal diseases that can cause foveal hypoplasia, so it remains to be seen whether the structure-function patterns observed in albinism can be generalized to individuals with foveal hypoplasia from other causes. Additionally, there is greater representation of participants with less severe foveal hypoplasia in this study, likely because subject factors associated with more severe foveal hypoplasia (such as visual acuity or nystagmus) can influence retinal imaging success.^45^ Finally, the cohort included both children and adults and so cannot provide any conclusions about differences between foveal structure or BCVA in early childhood versus adulthood. This may also limit our model's accuracy when using foveal structure in childhood to predict adult visual acuity, particularly because foveal structure is known to evolve at least until the age of three or four years old.^46^ Our relatively small sample size may limit how our findings may be generalized to the greater population with albinism. However, we find a similar trend between foveal hypoplasia grade and BCVA as that shown in a previous study with a smaller sample size,^2^ indicating that these trends likely represent a majority of the population. Finally, the predictive properties of the model presented here are retrospective statistical estimates. The model should be validated prospectively in a larger cohort, ideally in more homogenous groups assigned by age and genetic diagnosis (such as the recent study by Kuht et al.^47^), before the model is applied for clinical use.

## Conclusions

Foveal hypoplasia is an important predictor for BCVA in albinism. Although categorical grading is accessible and straightforward for clinicians, its predictive power is imperfect, and categorical grades do not describe the full spectrum of foveal specialization in albinism. Quantitative foveal structural metrics presented here address the limitations of existing quantitative metrics by normalizing retinal layer thickness measurements at the fovea to the parafovea, and they reveal greater variability in foveal structure than indicated by categorical grades. We also find that they account for more of the variance in BCVA in albinism than categorical foveal hypoplasia grade, although the improvement in predictive power is modest. Our results indicate that there are additional factors not included in this study that underly the variable BCVA in albinism.

## Supplementary Material

Supplement 1

Supplement 2

Supplement 3

Supplement 4

Supplement 5
