# Supplementary material for: Quantitative Foveal Structural Metrics as Predictors of Visual Acuity in Human Albinism
Source: Invest Ophthalmol Vis Sci. 2024 Mar 5;65(3):3. doi: 10.1167/iovs.65.3.3 (PMC10916884; doi:10.1167/iovs.65.3.3)
Supplement: Supplement 1 [file iovs-65-3-3_s001.pdf]

Supplementary Table S1: Participant cohort

Participant ID	Sex*	Age (years)	Genotype†	Albinism Status	Eye Used‡	Foveal Hypoplasia Grade	Visual Acuity (logMAR)§
JC_0103	M	16	*GPR143* c.797T>C, p.L266P (hemi; VUS)	Suspected	OD	3	0.60
JC_0131	M	20	*OCA2* c.2228C>T; p.P743L	Likely	OD	1b	0.28
JC_0150	F	21	No pathogenic variants detected	Suspected	OD	1b	0.56
JC_0156#	F	10	*TYR* c.575C>A, p.S192Y<br>*TYR* c.1118C>A, p.T373K	Likely	N/A	N/A	N/A
JC_0157#	M	12	*TYR* c.575C>A, p.S192Y<br>*TYR* c.1118C>A, p.T373K	Likely	N/A	N/A	N/A
JC_0456	M	14	*OCA2* c.1327G>A, p.V443I<br>*OCA2* c.1555_delG (fs)<br>*TYRP1* c.1103_delA	Confirmed	OS	1a	0.54
JC_0459	F	21	*OCA2* c.2339G>A, p.G780D (hom)	Confirmed	OS	3	0.68‖
JC_0492	F	28	*TYR* c.1147G>A, p.D383N<br>*TYR* c.1217C>T, p.P406L	Confirmed	OD	1b	0.14
JC_0494	M	9	*OCA2* c.868A>G, R290G<br>*OCA2* c.1465A>G, N489D	Confirmed	OS	2	0.60
KS_0551	M	30	*GPR143* c.112G>A, p.G38R (VUS)	Suspected	OS	2	0.68‖
JC_0598	M	9	*TYR* c.575C>A, S192Y (hom)<br>*OCA2* c.1327G>A, p.V443I	Likely	OD	1b	0.54‖
DC_0831	M	7	*TYR* c.575C>A, p.S192Y<br>*TYR* c.1205G>A, p.R402Q<br>*TYR* c.1467_1468insT (fs)	Likely	OD	1b	0.34‖
JC_10061	M	28	*OCA2* 2.7kb_del (hom)	Confirmed	OD	3	0.60
JC_10073	F	23	*TYR* c.976C>T, p.Q326* (hom)	Confirmed	OS	3	0.74
JC_10074	F	20	*OCA2* c.2207C>T, p.S736L (VUS)	Suspected	OD	2	0.56
JC_10081#	F	11	*OCA2* c.79G>A, p.G27R (hom; VUS)	Suspected	N/A	2	0.76
JC_10092	F	22	*TYR* c.575C>A, p.S192Y (hom)<br>*TYR* c.1118C>A, p.T373K<br>*TYR* c. 1205G>A, p.R402Q	Likely	OD	2	0.66
JC_10093	M	22	*TYR* c.1205G>A, p.R402Q (hom)<br>*GPR143* c.346T>G, p.C116G (hemi)	Confirmed	OS	2	0.56
TC_10110	F	50	*TYR* c.1205G>A, p.R402Q (hom)	Suspected	OS	1b	0.12

DC_10138[#]	M	7	*TYR* c.26T>C, p.L9P (VUS)<br>*TYR* c.980A>G, p.Y327C<br>*OCA2* c.1109T>C, p.I370T (VUS)	Likely	N/A	N/A	N/A
JC_10192	F	6	*TYR* c.575C>A, p.S192Y<br>*TYR* c.1205G>A, p.R402Q	Suspected	OD	1b	0.46
JC_10193	M	16	*OCA2* c.1327G>A, p.V443I (hom)	Confirmed	OD	1b	0.24
JC_10203	F	6	*TYR* c.575C>A, p.S192Y<br>*TYR* c.1205G>A, p.R402Q<br>*TYR* c.1467_1468insT (fs)	Likely	OD	2	0.62
JC_10227	F	18	*TYR* c.575C>A, p.S192Y<br>*OCA2* c.1327G>A, p.V443I	Likely	OD	2	0.32
JC_10228	F	15	*TYR* c.575C>A, p.S192Y<br>*OCA2* c.1327G>A, p.V443I	Likely	OS	1b	0.56
JC_10230	F	18	*TYR* c.575C>A, p.S192Y (hom)<br>*TYR* c.1205G>A, p.R402Q<br>*TYR* c.1265G>A, p.R422Q	Likely	OD	2	0.56
JC_10269	F	5	*TYR* c.575C>A, p.S192Y<br>*HPS6* c.108_109ins22bp (fs)	Likely	OS	2	0.84
JC_10278	M	14	*TYR* c.286_287insA (fs)<br>*TYR* c.575C>A, p.S192Y<br>*TYR* c.1205G>A, p.R402Q	Likely	OD	1b	0.18
JC_10279[#]	M	12	*TYR* c.832C>T, p.R278*<br>*TYR* c.929_930insC (fs)	Confirmed	N/A	N/A	N/A
JC_10287	M	10	*GPR143* exon4-9_del (hemi)	Confirmed	OD	2	0.50
KS_10314	F	5	*TYR* c.1A>G, p.M1V<br>*TYR* c.575C>A, p.S192Y<br>*TYR* c.1205G>A, p.R402Q (hom)	Likely	OD	2	0.44[‖]
JC_10496	M	69	*TYR* c.1205G>A, p.R402Q<br>*OCA2* c.1327G>A, p.V443I<br>*OCA2* c.1465A>G, p.N489D	Confirmed	OD	1b	0.32
JC_10508	F	37	*TYR* c.575C>A, p.S192Y<br>*TYR* c.1118C>A, p.T373K<br>*TYR* c.1205G>A, p.R402Q (hom)	Likely	OD	2	0.16
JC_10552	F	10	*OCA2* c.1842+2T>C (splice site)	Likely	OD	3	0.48
JC_10725	F	25	*TYR* c.338_339insAG (fs)<br>*TYR* c.368T>C, p.I123T	Confirmed	OD	2	0.60

JC_10726	M	23	*TYR* c.338_339insAG (fs)<br>*TYR* c.368T>C, p.I123T	Confirmed	OD	2	0.82
JC_10746#	F	20	*TYR* c.529G>A, p.V177I<br>*TYR* c.575C>A, p.S192Y<br>*TYR* c.1118C>A, p.T373K<br>*TYR* c.1205G>A, p.R402Q	Confirmed	N/A	N/A	N/A
JC_10797	M	17	*TYR* c.1205G>A, p.R402Q<br>*SLC45A2* c.443G>A, p.G148D (VUS)<br>*SLC45A2* c.665C>A, p.S222Y (VUS)	Suspected	OD	1b	0.46
JC_10841	M	25	*TYR* c.575C>A, p.S192Y<br>*TYR* c.1205G>A, p.R402Q<br>*HPS3* c.15C>G, p.Y5*<br>*HPS3* c.2464C>T, p.R822*	Confirmed	OS	2	0.72
BB_10965	F	44	*TYR* c.1205G>A, p.R402Q (hom)<br>*SLC24A5* c.1361T>A, p.L454* (hom)	Confirmed	OS	1b	0.24
GS_10977	F	18	*OCA2* c.1327G>A, p.V443I	Likely	OD	2	0.62
GS_10979	M	17	*TYR* c.575C>A, p.S192Y (hom)<br>*TYR* c.1205G>A, p.R402Q (hom)	Suspected	OD	1b	0.08
JC_11046	M	37	*TYR* c.575C>A, p.S192Y<br>*OCA2* c.1327G>A, p.V443I	Likely	OS	2	0.66
JC_11084#	M	54	*TYR* c.1A>G, p.M1V<br>*TYR* c.242C>T, p.P81L<br>*TYR* c.575C>A, p.S192Y<br>*TYR* c.1205G>A, p.R402Q	Confirmed	N/A	2	1.06
GS_11148	F	9	*TYR* c.575C>A, p.S192Y<br>*TYR* c.1147G>A, p.D383N<br>*TYR* c.1205G>A, p.R402Q (hom)	Likely	OD	1b	0.24
JC_11430	F	16	*TYR* c.575C>A, p.S192Y<br>*TYR* c.1205G>A, p.R402Q<br>*TYR* c.1423_1433del11bp (fs)	Likely	OD	2	0.28
DC_11476	M	17	*TYR* c.1205G>A, p.R402Q<br>*BLOC1S3* c.301_302insC (hom)	Confirmed	OD	4	0.88
DC_11599#	F	6	*TYR* c.575C>A, p.S192Y<br>*TYR* c.1118C>A, p.T373K<br>*TYR* c.1205G>A, p.R402Q<br>*TYR* c.1430_1442del13bp (fs)	Confirmed	N/A	N/A	N/A

GS_11807	M	37	*TYR* c.575C>A, p.S192Y (hom)<br>*GPR143* exon1-2_del (hemi)	Confirmed	OS	3	0.68
JC_11817[#]	F	23	*TYR* c.575C>A, p.S192Y (hom)<br>*TYR* c.1118C>A, p.T373K (hom)	Confirmed	N/A	N/A	N/A
JC_11822	F	40	*TYR* c.575C>A, p.S192Y<br>*TYR* c.729_730delGT (fs)<br>*TYR* c.1118C>A, p.T373K	Confirmed	OD	2	0.74
JC_11824	F	12	*TYR* c.823G>T, p.V275F<br>*TYR* c.1336G>A, p.G446S	Confirmed	OS	3	0.54
JC_11825[#]	M	12	*TYR* c.823G>T, p.V275F<br>*TYR* c.1336G>A, p.G446S	Confirmed	N/A	N/A	N/A
AD_11837	M	17	*TYR* c.575C>A, p.S192Y<br>*TYR* c.823G>T, p.V275F<br>*TYR* c.1205G>A, p.R402Q	Likely	OD	1b	0.16
JC_11841[#]	M	14	No data	Suspected	N/A	N/A	N/A
JC_11842[#]	F	10	*TYR* c.71G>A, p.C24Y (VUS)<br>*TYR* c.230G>A, p.R77Q	Likely	N/A	N/A	N/A
JC_11849	F	25	*TYR* c.575C>A, p.S192Y (hom)<br>*SLC45A2* c.3G>A, p.M1I<br>*SLC45A2* c.1082T>C, p.L361P (VUS)	Likely	OS	2	0.54
JC_11850	M	33	*TYR* c.71G>A, p.C24Y (VUS)<br>*TYR* c.707G>C, p.W236S<br>*TYR* c.1205G>A, p.R402Q	Likely	OS	4	0.88
JC_11851	F	37	*TYR* c.575C>A, p.S192Y<br>*TYR* c.1101C>A, p.H367Q<br>*TYR* c.1118C>A, p.T373K	Confirmed	OD	2	0.72
JC_11854	F	33	*OCA2* c.373_374delGA (fs)	Likely	OD	2	0.64
JC_11895[#]	M	15	*TYR* c.896G>A, p.R299H (hom)	Confirmed	N/A	N/A	N/A
JC_11896	F	13	*OCA2* c.1349C>T, p.T450M<br>*OCA2* c.1363A>G, p.R455G (VUS)<br>*OCA2* c.1994C>T, p.A665V (VUS)	Likely	OS	2	0.52
AD_11897	M	49	*TYR* c.823G>T, p.V275F<br>*TYR* c.1111A>T, p.N371Y	Confirmed	OS	4	0.64

JC_11898	F	30	*TYR* c.575C>A, p.S192Y<br>*OCA2* c.1327G>A, p.V443I<br>*OCA2* c.1983G>T, p.L661F (VUS)	Likely	OS	2	0.58
JC_11899[#]	F	13	*TYR* c.575C>A, p.S192Y<br>*TYR* c.1205G>A, p.R402Q (hom)<br>*OCA2* c.632C>T, p.P211L<br>*OCA2* c.1465A>G, p.N489D	Confirmed	N/A	N/A	N/A
JC_11900	F	15	*TYR* c.575C>A, p.S192Y<br>*TYR* c.1205G>A, p.R402Q (hom)<br>*OCA2* c.632C>T, p.P211L<br>*OCA2* c.1465A>G, p.N489D	Confirmed	OS	2	0.56
GS_11902	M	16	*TYR* c.575C>A, p.S192Y<br>*GPR143* c.853A>T, p.R285* (hemi)	Confirmed	OD	3	0.64
AD_11925	M	22	*TYR* c.650G>A, p.R217Q<br>*TYR* c.823G>T, p.V275F<br>*TYR* c.1205G>A, p.R402Q	Confirmed	OS	2	0.58
JC_11934	M	10	*OCA2* c.163delG (fs)<br>*HPS5* c.253C>T, p.H85Y (VUS)	Likely	OS	2	1.00
SS_11938	F	22	*OCA2* c.1327G>A, p.V443I	Suspected	OD	1b	0.14
SS_11939	M	35	*TYR* c.650G>A, p.R217Q<br>*TYR* c.823G>T, p.V275F<br>*TYR* c.1205G>A, p.R402Q	Confirmed	OD	2	0.58
AD_11940	F	16	*TYR* c.-4586_del4461bp_insTT<br>*TYR* c.880G>A, p.E294K<br>*TYR* c.1205G>A, p.R402Q	Confirmed	OS	2	0.44
AD_11941	M	11	*TYR* c.613C>A, p.P205T<br>*TYR* c.1217C>T, p.P406L	Confirmed	OS	1b	0.34
AD_11991	M	9	*TYR* c.1205G>A, p.R402Q (hom)<br>*GPR143* c.-171G>A (hemi; VUS)	Suspected	OD	1b	0.80
JC_11992	M	14	*TYR* c.575C>A, p.S192Y (hom)<br>*OCA2* c.1465A>G, p.N489D<br>*OCA2* c.2330G>A, p.C777Y	Confirmed	OD	2	0.46
AD_12015	M	46	*TYR* c.575C>A, p.S192Y	Suspected	OD	1b	0.52
AD_12016[#]	F	13	*TYR* c.575C>A, p.S192Y<br>*TYR* c.1205G>A, p.R402Q	Suspected	OS	2	0.74

AD_12021	M	11	*TYR* c.575C>A, p.S192Y<br>*TYR* c.1205G>A, p.R402Q<br>*OCA2* c.1327G>A, p.V443I<br>*HPS3* c.1136C>T, p.T379M (VUS)	Likely	OS	1b	0.48
AD_12090	F	19	*TYR* c.1118C>A, p.T373K<br>*TYR* c.1205G>A, p.R402Q	Likely	OS	1b	0.44
AD_12112	F	10	*TYR* c.575C>A, p.S192Y<br>*TYR* c.823G>T, p.V275F<br>*TYR* c.1205G>A, p.R402Q	Likely	OD	2	0.74
SS_12121	F	5	*TYR* c.575C>A, p.S192Y (hom)<br>*OCA2* c.1255C>T, p.R419W<br>*TYRP1* c.785C>T, p.T262M (VUS)	Likely	OS	1a	0.66
DC_12122	F	8	*OCA2* c.1025A>G, p.Y342C<br>*OCA2* c.1465A>G, p.N489D	Confirmed	OD	1a	0.42
SS_12124	M	9	*TYR* c.1205G>A, p.R402Q (hom)<br>*TYRP1* c.140_141delCT (fs)<br>*TYRP1* c.194G>A, p.C65Y (VUS)	Confirmed	OD	1b	0.56
JC_12260	F	48	*TYR* c.575C>A, p.S192Y<br>*TYR* c.823G>T, p.V275F<br>*TYR* c.1205G>A, p.R402Q	Confirmed	OS	2	0.16
JC_12261#	M	9	*TYR* c.823G>T, p.V275F (hom)<br>*OCA2* c.1327G>A, p.V443I	Confirmed	N/A	N/A	N/A
JC_12500	F	26	*TYR* c.575C>A, p.S192Y<br>*TYR* c.1118C>A, p.T373K<br>*TYR* c.1147G>A, p.D383N	Confirmed	OS	3	0.72
JC_12501	M	30	*TYR* c.976C>T, p.Q326* (hom)	Confirmed	OD	4	0.82
JC_12522	F	22	*SLC45A2* c.310C>T, p.P104S<br>*SLC45S2* c.606G>C, p.W202C	Confirmed	OD	2	0.74
JC_12536	M	14	*TYR* c.575C>A, p.S192Y<br>*TYR* c.1205G>A, p.R402Q<br>*GPR143* exon 1 del (hemi)	Confirmed	OS	4	0.72
JC_12537	M	75	*TYR* c.575C>A, p.S192Y<br>*GPR143* exon 1 del (hemi)	Confirmed	OD	3	0.64

*M = male, F = female

[†]fs = frameshift, hemi = hemizygous, hom = homozygous, kb = kilobases. All listed mutations are presumed to be pathogenic, except *TYR* p.S192Y, *TYR* p.R402Q, and any mutations noted as a variants of unknown significance (VUS).
[‡]OD = right eye, OS = left eye, N/A = not applicable
[§]logMAR = logarithm of minimum angle of resolution
[||]Visual acuity with correction; all other visual acuity measurements are best-corrected visual acuity.
[#]Participants were excluded from final analyses due to poor OCT image quality.
