# Supplementary material for: Quantitative Foveal Structural Metrics as Predictors of Visual Acuity in Human Albinism
Source: Invest Ophthalmol Vis Sci. 2024 Mar 5;65(3):3. doi: 10.1167/iovs.65.3.3 (PMC10916884; doi:10.1167/iovs.65.3.3)
Supplement: Supplement 2 [file iovs-65-3-3_s002.pdf]

Supplementary Table S2: Novel presumed albinism-causing variants

Gene	Variant	Subject	Evidence of Pathogenicity*	ACMG Classification
TYR	c.338_339insAG (fs)	JC_10725<br>JC_10726	PVS1, PM2, PP4	Pathogenic
TYR	c.529G>A, p.V177I	JC_10746	PM2, PM5, PP3, PP4	Likely pathogenic
TYR	c.1430_1442del13bp (fs)	DC_11599	PVS1, PM2, PP4	Pathogenic
OCA2	c.373_374delGA (fs)	JC_11854	PVS1, PM2, PP4	Pathogenic
TYRP1	c.140_141delCT (fs)	SS_12124	PVS1, PM2, PP4	Pathogenic
SLC45A2	c.3G>A, p.M1I	JC_11849	PM1, PM2, PP3, PP4	Likely pathogenic
GPR143	c.853A>T, p.R285*	GS_11902	PVS1, PM2, PP4	Pathogenic

*PVS = very strong evidence of pathogenicity; PM = moderate evidence of pathogenicity; PP = supporting evidence of pathogenicity. For a description of each numbered subtype of evidence of pathogenicity, see Richards et al.[1]

1. Richards S, Aziz N, Bale S, et al. Standards and guidelines for the interpretation of sequence variants: A joint consensus recommendation of the American College of Medical Genetics and Genomics and the Association for Molecular Pathology. *Genet Med* 2015;17:405-424.
