# Supplementary material for: Quantitative Foveal Structural Metrics as Predictors of Visual Acuity in Human Albinism
Source: Invest Ophthalmol Vis Sci. 2024 Mar 5;65(3):3. doi: 10.1167/iovs.65.3.3 (PMC10916884; doi:10.1167/iovs.65.3.3)
Supplement: Supplement 3 [file iovs-65-3-3_s003.pdf]

Supplementary Appendix 1

Venetic variants were evaluated for pathogenicity and novelty by comparing them to the National Center for Biotechnology Information ClinVar database (http://ncbi.nlm.nih.gov/clinvar) and the Albinism Database (http://albinismdb.med.umn.edu). Variants were considered to be albinism-causing if the consensus of reports in ClinVar was either “pathogenic” or “likely pathogenic”. Novel variants that were not reported in ClinVar, as well as those that had no clear consensus in ClinVar, were evaluated for pathogenicity using the American College of Medical Genetics and Genomics (ACMG) guidelines for interpretation of variants.[1] These variants were considered albinism-causing if the ACMG classification standards indicated they were either “pathogenic” or “likely pathogenic.” Computational evidence for pathogenicity was assessed using SIFT (http://sift.bii.a-star.edu.sg),[2] PROVEAN (http://provean.jcvi.org),[3] and PolyPhen-2 (http://genetics.bwh.harvard.edu/pph2).[4] Participants were considered to have a confirmed genetic diagnosis if they had two albinism-causing variants in an OCA or HPS gene (autosomal recessive inheritance), a hemizygous albinism-causing variant in *GPR143* (X-linked recessive inheritance), or a single albinism-causing variant in *TYR* that was known to be in trans with the hypomorphic *TYR* alleles S192Y and R402Q.[5,6]

Supplementary Appendix 1 Bibliography

1. Richards S, Aziz N, Bale S, et al. Standards and guidelines for the interpretation of sequence variants: A joint consensus recommendation of the American College of Medical Genetics and Genomics and the Association for Molecular Pathology. *Genetics in Medicine* 2015;17:405-424.
2. Sim NL, Kumar P, Hu J, Henikoff S, Schneider G, Ng PC. SIFT web server: Predicting effects of amino acid substitutions on proteins. *Nucleic Acids Research* 2012;40:W452-457.
3. Choi Y, Chan AP. PROVEAN web server: A tool to predict the functional effect of amino acid substitutions and indels. *Bioinformatics* 2015;31:2745-2747.
4. Adzhubei I, Jordan DM, Sunyaev SR. Predicting functional effect of human missense mutations using PolyPhen-2. *Current Protocols in Human Genetics* 2013;Chapter 7:1-41.
5. Norman CS, O'Gorman L, Gibson J, et al. Identification of a functionally significant tri-allelic genotype in the Tyrosinase gene (TYR) causing hypomorphic oculocutaneous albinism (OCA1B). *Scientific Reports* 2017;7:4415.
6. Lin S, Sanchez-Bretaño A, Leslie JS, et al. Evidence that the Ser192Tyr/Arg402Gln in cis Tyrosinase gene haplotype is a disease-causing allele in oculocutaneous albinism type 1B (OCA1B). *NPJ Genomic Medicine* 2022;7:2.
