# Supplementary material for: Quantitative Foveal Structural Metrics as Predictors of Visual Acuity in Human Albinism
Source: Invest Ophthalmol Vis Sci. 2024 Mar 5;65(3):3. doi: 10.1167/iovs.65.3.3 (PMC10916884; doi:10.1167/iovs.65.3.3)
Supplement: Supplement 4 [file iovs-65-3-3_s004.pdf]

Supplementary Appendix 2

We used ROC analyses (ROC curves shown below in **Figure S1**) to determine whether our quantitative metrics could objectively differentiate between the presence or absence of specific foveal specializations. Using subjective foveal hypoplasia grades as ground truth, we compared IRL ratio between participants with a foveal depression (grades 1a and 1b) and those without (grades 2-4); then we compared OS ratio between participants with OS elongation (grades 1a-2) and those without (grades 3-4); and finally we compared ONL ratio between participants with ONL thickening (grades 1a-3) and those without (grade 4). The area under the curve (AUC) for IRL ratio was 0.98 ($p < 0.0001$) and the optimum cutoff value was 1.175 (sensitivity = 0.92, specificity = 0.92). The AUC for OS ratio was 0.88 ($p < 0.0001$) and the optimum cutoff value was 1.375 (sensitivity = 0.93, specificity = 0.75). The AUC for ONL ratio was 0.82 ($p = 0.018$) and the optimum cutoff value was 1.075 (sensitivity = 0.80, specificity = 0.80).

We then used these quantitative cutoff values to assign foveal hypoplasia grades objectively. For each participant we first assessed the presence or absence of OS elongation based on OS ratio. For participants who had OS elongation (i.e. OS ratio > 1.375), we assessed the presence or absence of IRL excavation based on IRL ratio; those with IRL ratios < 1.175 were assigned grade 2, and those with IRL ratios > 1.175 were assigned grade 1a-1b. For participants who did not have OS elongation (i.e. OS ratio < 1.375), we assessed the presence or absence of ONL thickening based on ONL ratio; those with ONL ratios < 1.075 were assigned grade 4, and those with ONL ratios > 1.075 were assigned grade 3. Objective foveal hypoplasia grades agreed with subjective grades in 47 participants (63.5%), which corresponded to moderate agreement between methods (weighted Cohen's $\kappa = 0.605$).

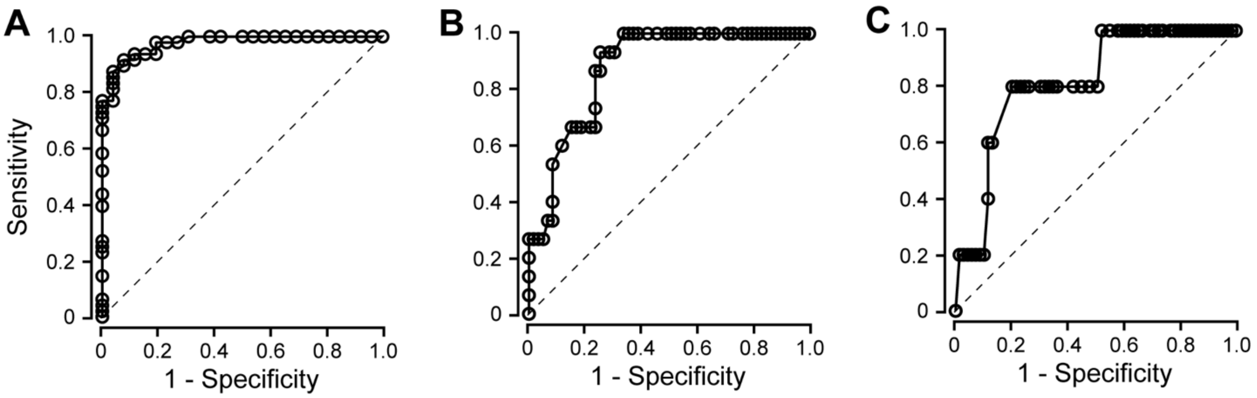

Figure S1: ROC curves demonstrate that IRL ratio (A) can quantitatively differentiate between foveal hypoplasia grades with and without a foveal depression (i.e., grades 1a-1b and 2-4, respectively), that OS ratio (B) can quantitatively differentiate between those with and without OS elongation (i.e., grades 1a-2 and 3-4, respectively), and that ONL ratio (C) can quantitatively differentiate between those with and without ONL thickening (i.e., grades 1a-3 and 4, respectively). Open circles show the results of the ROC analysis for each metric with solid line segments connecting each point; dashed lines show where sensitivity = 1 – specificity, which corresponds to performance equivalent to random chance.
