# Supplementary material for: Quantitative Foveal Structural Metrics as Predictors of Visual Acuity in Human Albinism
Source: Invest Ophthalmol Vis Sci. 2024 Mar 5;65(3):3. doi: 10.1167/iovs.65.3.3 (PMC10916884; doi:10.1167/iovs.65.3.3)
Supplement: Supplement 5 [file iovs-65-3-3_s005.pdf]

Supplementary Appendix 3

We compared the predictive power of our combined linear model to that of previously-published quantitative metrics of foveal structure (Rufai et al., *Ophthalmol* 2019). These metrics include OS length, photoreceptor (PR) length, and the foveal developmental index (FDI), all of which measure retinal thickness only at the incipient fovea. PR length was defined as the distance between the outer OPL boundary and the IZ at the fovea. FDI was defined as the PR length divided by the total retinal thickness (distance between the ILM and RPE) at the fovea. These measurements were performed using the DOCTRAP segmentation of the Bioptigen OCT scans.

The goodness of fit for linear predictive models of BCVA based on each of these foveal metrics is shown below in **Supplementary Table S3**. All predictors had worse goodness of fit and inferior correlation between predicted and observed BCVA than the combined linear model based on IRL ratio, OS ratio, ONL ratio, participant sex, and age (see **Table 1**). PR length and FDI also had lower predictive power than categorical grade. Notably, OS length had slightly higher predictive power than categorical grade.

Supplementary Table S3: Cross-validated goodness of fit for fovea-only quantitative metrics

Foveal Predictor	Adjusted R^2	RMSE*	Correlation (*R*) between predicted and observed BCVA
OS length	0.394	0.164	0.632
PR length	0.328	0.172	0.581
FDI	0.277	0.180	0.535

*RMSE = Root mean squared error
